# *Synchroamedogensis*, a new species of Synchroidae (Coleoptera) from Xizang, China

**DOI:** 10.3897/BDJ.12.e122792

**Published:** 2024-05-23

**Authors:** Zhao Pan, Shaopeng Wang

**Affiliations:** 1 School of Life Sciences, Hebei University, Baoding City, China School of Life Sciences, Hebei University Baoding City China

**Keywords:** *Synchroa*, new species, Tibet, China

## Abstract

**Background:**

*Synchroamedogensis*, a new species of Synchroidae Lacordaire, 1859, is described and illustrated, based on a single male collected from Mêdog, Xizang, China. This new species is close to *S.elongatula* Nikitsky, 1999 and *S.melanotoides* Lewis, 1895, but could be distinguished by the elongated antennae and elytra, the apically narrowed prosternal process and the stout parameres.

**New information:**

The new information of this new species provided in this paper include: description, type information, distribution and comparison amongst *S.medogensis* sp. nov., *S.elongatula* and *S.melanotoides*.

## Introduction

Synchroidae Lacordaire, 1859 is a small beetle family of Tenebrionoidea and has been recently revised (Nikitsky 1999, Hsiao 2015, Hsiao et al. 2016, Hsiao et al. 2018). It only has ten extant species belonging to four genera and is widely distributed throughout the Oriental, Palaearctic and Nearctic Regions (Konvička and Hsiao 2018). The genus *Synchroa* Newman, 1838 includes six species, five distributed in China and its surrounding areas (Hsiao et al. 2018, Konvička and Hsiao 2018). Adults are distinguished from other genera by the combination of the following characters: compound eyes middle in size, interocular space approximately 1.5–1.7× as wide as eye diameter; pronotum with complete lateral carinae; procoxae distinctly separated; pretarsal claws simple, without teeth along ventral margin; median lobe of aedeagus elongate, slender and tapered (Hsiao et al. 2018).

In May 2023, a new *Synchroa* species was discovered in Mêdog County of Xizang, China. It is described and illustrated below.

## Materials and methods

The holotype has been deposited at the Museum of Hebei University, Baoding, China (MHBU). The specimen was observed using a Nikon SMZ1500 and the images were taken with a Canon EOS 5D Mark III (Canon Inc., Tokyo, Japan) connected to a Laowa FF 100 mm F2.8 CA-Dreamer Macro 2× or Laowa FF 25 mm F2.8 Ultra Macro 2.5-5× (Anhui Changgeng Optics Technology Co., Ltd, Hefei, China). Label data are presented verbatim. Line breaks on labels are denoted by a slash (/); metadata and notes (not written on the labels themselves) are presented in square brackets ([]). Most of the terms in the description are from previous literature (e.g. Hsiao et al. (2018), Konvička and Hsiao (2018)).

## Taxon treatments

### Synchroa medogensis

Pan & Wang sp. nov.

A8EC6E69-5D86-5FC2-ACAF-32776B8C9B1A

4459BE6E-C519-4171-B57D-86BEB10C6F83

#### Materials

- **Type status:** Holotype. **Occurrence:** recordedBy: Xinglong Bai; sex: male; lifeStage: adult; occurrenceID: 48E1E27E-7DBF-5D5C-9847-20D2253E3607; **Taxon:** kingdom: Animalia; phylum: Arthropoda; class: Insecta; order: Coleoptera; family: Synchroidae; genus: Synchroa ; taxonRank: species; verbatimTaxonRank: sp.; scientificNameAuthorship: Pan & Wang; nomenclaturalCode: ICZN; taxonomicStatus: accepted; nomenclaturalStatus: nov. sp.; **Location:** country: China; countryCode: China/CN; stateProvince: Xizang; county: Mêdog; locality: Baibung; verbatimElevation: 754 m; verbatimCoordinateSystem: decimal degrees; decimalLatitude: 29.235938; decimalLongitude: 95.168368; geodeticDatum: GCJ02; **Identification:** identifiedBy: Pan Z; Wang S-P; dateIdentified: 2024; **Record Level:** language: en; basisOfRecord: PreservedSpecimen

#### Description

**Figure 1.**
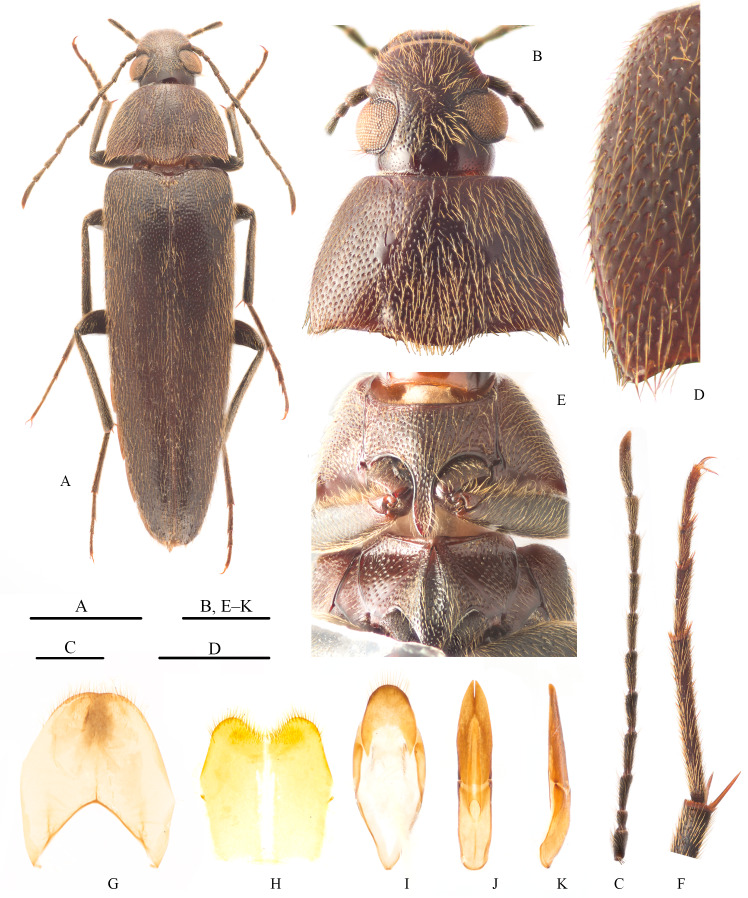
*Synchroamedogensis* sp. nov., male, holotype. **A** habitus, dorsal view; **B** head and pronotum, dorsal view; **C** antenna, right; **D** left side of pronotum, dorsolateral view; **E** pro- and mesosternum, ventral view; **F** metatibial apex and spurs and metatarsi, lateral view; **G** abdominal tergite VIII, dorsal view; **H** abdominal sternite VIII, ventral view; **I** abdominal sternite IX and fused tergite IX and X, dorsal view; **J–K** aedeagus: **J** dorsal view; **K** lateral view. Scale bars: 5 mm (A); 0.05mm (D); 1 mm (others).

Male: body (Fig. 1A) shiny, completely reddish-brown, antennae and legs darker. Body elongate, slightly flattened, elytra strongly narrowed posteriorly. Body covered with yellowish decumbent long setae. Body length 16.0 mm, width (at humerus of elytra) 4.0 mm.

Head (Fig. 1B) approximately as long as wide; punctation irregular, larger and denser basally and smaller and sparser apically; interspaces amongst punctures smooth. Eyes prominent, protruding from margin of head, interocular space approximately 1.5× as wide as eye diameter. Vertex with longitudinal furrows along inner margin of each eye. Last maxillary palpomere subsecuriform. Antennae (Fig. 1C) filiform, extending back to humerus of elytra, with 11 antennomeres; all antennomeres cylindrical and longer than wide; antennomere I slightly widened at apex, wider than other antennomeres; II shortest; XI longest, approximately 5.7× as long as maximum width; length ratios of antennomeres I–XI: 1.51 : 1.00 : 1.79 : 2.17 : 2.00 : 2.08 : 1.94 : 2.01 : 1.92 : 1.88 : 3.33.

Pronotum (Fig. 1B) approximately 1.5× as wide as long, widest near base; punctures with similar size to that on head, gradually denser towards lateral sides; basal angles distinct, slightly projecting; anterior margin almost straight; lateral margins rounded and narrowing anteriad, distinctly bordered at basal 1/3 to 1/4 (Fig. 1D); posterior margin bisinuate, with obtuse median lobe; disc with two subrounded impunctate smooth areas on sides of centre, one large shallow depression at centre of base and two deep depressions on sides of base. Elytra elongate, nearly 2.9× as long as wide, as wide as pronotal width at humeri, narrowed posteriorly, apex rounded; disc covered with oval punctures, denser in lateral and basal part; interspaces wider than puncture diameter. Prosternal process long, bordered laterally, narrowed apically (Fig. 1E). Mesosternum with shallow, oval mesosternal cavity, moderately punctate (Fig. 1E). Legs slender; all tibiae and meso- and metatarsomere I with pectinate teeth along apical margin (Fig. 1F); tibiae with 2 spurs at apex, spurs with micro-teeth along ventral margins (Fig. 1F), metatibial inner spur approximately 0.3× as long as metatarsomere I (Fig. 1F); tarsomeres simple, length ratio of metatarsomeres as follows: 4.31: 1.87: 1.00: 1.56 (Fig. 1F); pretarsal claws simple, ventral margin smooth (Fig. 1F).

Abdominal ventrite V subtruncate, with lateral sides nearly straight to slightly rounded, slightly emarginate in middle of posterior margin; tergite VIII (Fig. 1G) without median strut at apex, slightly emarginate in middle of posterior margin, moderately pubescent apically; sternite VIII (Fig. 1H) concave in middle of posterior margin, forming two roundly angular lobes on both sides, moderately pubescent apically; sternite IX (Fig. 1I) without *spiculum gastrale* on apex; tergite IX and X completely fused, posterior margin rounded and moderately pubescent (Fig. 1I). Aedeagus (Fig. 1J–K). Lanceolate; phallobase curved dorsally, lateral sides almost straight and subparallel on apical half, gradually narrowed basically; in dorsal view, parameres stout, approximately 1.1× as long as phallobase and approximately 2.75× as long as its maximum width, basal 5/6 fused, lateral sides slightly widened medially, gradually narrowed apically; median lobe elongate, tapered, strongly narrowed in apical half.

Female. Unknown.

#### Diagnosis

This new species resembles *Synchroaelongatula* Nikitsky, 1999 (from Vietnam and Laos) and *S.melanotoides* Lewis, 1895 (from Russia, Japan, Korea and China) in the pronotal configuration with lateral margins bordered in posterior 1/3 to 1/4. Their differential diagnosis is summarised in Table 1.

#### Etymology

The name of this new species refers to its type locality, Mêdog County (Xizang, China).

#### Distribution

China: SE Xizang.

## Supplementary Material

XML Treatment for Synchroa medogensis

## Figures and Tables

**Table 1.** Diagnosis characters amongst *S.medogensis* sp. nov., *S.elongatula* and *S.melanotoides*

	***S.medogensis* sp. nov.**	***S.elongatula***	***S.melanotoides***
Male antennae	Elongate (Fig. 1C); antennomere XI ca. 5.7× as long as maximum width and 3.33× as long as II; IV–X distinctly longer than I, respectively; III slightly shorter than V.	Slightly shorter; antennomere XI ca. 3.0× as long as maximum width, and 2.20× as long as II; IV–X ca. as long as I, respectively; III distinctly longer than V.	Slightly shorter; antennomere XI ca. 3.1–4.1× as long as maximum width and 2.85× as long as II; IV–X distinctly longer than I, respectively; III distinctly longer than V.
Elytra	Elongate and slender, nearly 2.9× as long as wide (Fig. 1A)	Elongate and slender, ca. 3.0× as long as wide (fig. 4E in Hsiao et al. (2018))	Stout, ca. 2.4–2.6× as long as wide (fig. 4B in Hsiao et al. (2018))
Prosternal process	Narrowed apically (Fig. 1E)	Narrowed apically	Long oval apex
Male aedeagus	Parameres almost as long as phallobase, wider, ca. 2.75× as long as its maximum width; lateral margins gradually narrowed apically (Fig. 1J)	Parameres distinctly longer than phallobase, ca. 3.2× as long as its maximum width; lateral margins subparallel or sinuate (fig. 6M in Hsiao et al. (2018))	Parameres distinctly longer than phallobase, ca. 3.0–3.9× as long as its maximum width; lateral margins gradually narrowed apically and abruptly strongly narrowed at apex (fig. 6F in Hsiao et al. (2018))

## References

[B11224819] Hsiao Y. (2015). A new species of the genus *Synchroa* from Taiwan, with a key to the world fauna (Coleoptera: Synchroidae. Acta Entomologica Musei Nationalis Pragae.

[B11224828] Hsiao Y., Li Y., Liu Z., Pang H. (2016). A new species of *Synchroa* Newman from China (Coleoptera: Synchroidae. Zootaxa.

[B11224801] Hsiao Yun, Konvička Ondřej, Ko Chiun-Cheng (2018). The world fauna of Synchroidae Lacordaire, 1859 (Coleoptera, Tenebrionoidea, Synchroidae). European Journal of Taxonomy.

[B11224846] Konvička O., Hsiao Y. (2018). A description of *Synchroaruzzieri* sp. nov. from China (Coleoptera: Tenebrionoidea: Synchroidae) with a key to the world fauna of Synchroidae. Studies and Reportes Taxonomical Series.

[B11224873] Nikitsky N. B. (1999). To the knowledge of beetles of the family Synchroidae (Coleoptera, Tenebrionoidea) in the world fauna. Entomological Review.

